# Relationship between oral health literacy of caregivers and the oral health-related quality of life of children: a cross-sectional study

**DOI:** 10.1186/s12955-022-02019-4

**Published:** 2022-07-30

**Authors:** Sofia Rafaela Maito Velasco, Caroline Moraes Moriyama, Marcelo Bonecker, Luciane Butini, Jenny Abanto, José Leopoldo Ferreira Antunes

**Affiliations:** 1 Public Health School, University of São Paulo, São Paulo, Av. Dr. Arnaldo, 715 - Cerqueira César, São Paulo, SP 01246-904 Brazil; 2 Department of Paediatric and Orthodontic Dentistry, University of Sao Paulo, São Paulo, SP Brazil; 3 Department of Postgraduation Program in Dentistry, Metropolitana de Santos University, Santos, SP Brazil; 4 Oral Radiology in the School of Dentistry, School of Dentistry, São Leopoldo Mandic, Campinas, SP Brazil

**Keywords:** Oral health literacy, Oral health-related quality of life, Dental caries, Preschool-age children

## Abstract

**Background:**

Oral health literacy is the degree to which individuals have the capacity to obtain, process, and understand basic health information and services needed to make appropriate oral health decisions. However, scientific evidence about the oral health literacy of caregivers and the children’s oral health-related quality of life. The purpose of this study was to verify the relationship between the level of oral health literacy of caregivers and the children's oral health-related quality of life (OHRQOL).

**Methods:**

This study was conducted with children aged 2 to 4 in Diadema, São Paulo, Brazil. Six hundred thirty children were examined to assess the prevalence of dental caries (dmft index). Parents were interviewed to obtain sociodemographic status, oral conditions, and oral health literacy (OHL). The variable outcome was the children's OHRQOL as assessed by the Early Childhood Oral Health Impact Scale (ECOHIS). We fitted zero-inflated negative binomial regression (ZINB) models to evaluate associations between the study outcome and covariates in terms of PR (Prevalence Ratios), RR (Rate Ratios), and their respective Confidence Intervals (95% CI).

**Results:**

Children's OHRQOL was not associated with OHL. Dental caries had a negative impact on the children's quality of life (*p* < 0.05). A reduced impact on OHRQOL is also associated with having siblings (PR = 0.70, 95% CI 0.52–0.95). A higher age of the mother reduced OHRQOL impacts (PR = 0.72, 95% CI 0.52–0.98).

**Conclusions:**

The factors associated with children's OHRQOL were the number of siblings, the mothers' age, and dental caries. This study observed no association between parental OHL and children's OHRQOL.

## Background

Vulnerability to dental caries in preschool children has been associated with family- and parent-related factors [1–4]. Although it is recognized that caregivers play a critical role in the prevention and management of children's oral health status [5].

Oral health literacy (OHL) is defined as “the extent to which individuals are able to obtain, process, and understand basic oral health information and the services necessary for them to make appropriate health decisions” [6].

OHL is a topic of growing interest in the literature, with many studies having been conducted on this subject during the last decade [7–9]. However, there is a scarcity of published studies evaluating the impact of the literacy levels of parents and caregivers and oral health outcomes in the child population, such as caries and oral health-related quality of life (OHRQOL) in Brazil and worldwide. Some studies reported a correlation between high OHL of the parents and a lower prevalence of dental caries among children [10, 11].

Studies on OHRQOL assume that normative clinical oral health indicators do not immediately reflect how much people feel affected by their oral health conditions. In this regard, it has been observed that low OHL levels are also associated with worse OHRQOL [12, 13], which makes it even more relevant to assess the relationship between this condition with other oral health outcomes. Based on these findings, we hypothesized that the parents’ and caregivers’ OHL might affect their children's OHRQOL, on the one hand, and, on the other, influence their perception of the oral impacts related to the children and their families. In this respect, a single study carried out in the United States found a strong correlation between caregivers’ OHL and their preschool children’s OHRQOL [14]. The magnitude of this association remains uncertain, and precise knowledge of this information can contribute to the proper planning of programs and policies for promoting oral health.

This study aimed to assess the impact of preschool-age children’s OHRQOL and its association with the prevalence of dental caries and OHL of their parents and caregivers, also accounting the role of other characteristics of interest, such as sugar consumption, oral hygiene practices, socioeconomic conditions, and demographic factors as potential confounding variables.

## Methods

The project was approved by the Research Ethics Committee (68445317.3.0000.0075) of the University of São Paulo (USP) Faculty of Public Health. The participants in the study were preschool-age children from the city of Diadema, State of São Paulo, Brazil. Their legal guardians consented to both the children’s and their participation in the study and signed an informed consent form.

### Calculation of sample size and sample selection

To estimate the minimum size of a representative sample of children aged 2 to 4 years, the following assumptions were made: a standard error of 5%; a caries prevalence of 20.3%, according to the latest (2012) epidemiological survey in Diadema for the target age group [15]; a design effect of 2.0; and a significance level of 95%. Thus, a minimum sample size of 497 children was obtained. A 20% non-response rate was added to this value, getting a final sample size of 597 children and their respective parents or caregivers.

### Data collection

Dentist examiners collected all data, one for each of the 18 primary public health clinics of the municipality of Diadema, in September 2017, a neighboring town of São Paulo, the largest city in Brazil, which is affected by intense health and socioeconomic inequalities, despite being among the better-off cities in the country. For convenience, data gathering occurred during the National Campaign for Multiple Vaccination of Children. This methodological strategy has been employed and described in previous cross-sectional and trend studies in the same municipality and target age group [16–19]. To assess dental caries, each examiner collected data from 36 children (12 children in each of the three age groups that make up the sample).

### Training and calibration of examiners, note-takers, and assistants

Eighteen examining dentists underwent training and calibration for the clinical examination of dental caries. The training and calibration exercises were carried out in two sessions of four hours each. Clinical photographs were exhibited, and teeth gathered by the School of Dentistry from the University of São Paulo were used. Also, the various criteria for dental caries used in the study were explained.

Oral health assistants and community health agents received two training sessions of four hours each, with a 1-week break between them. The survey questionnaires and how they should be applied were presented and explained during these sessions.

### Questionnaires

The frequency of sugar consumption between meals (discrete variable, ≤ 3 or > 3 times/day) [20] and the frequency of daily toothbrushing (< 2 or ≥ 2 times/day) were evaluated.

The parents also answered a questionnaire on demographic and socioeconomic conditions: child's sex (female or male), child's age (1, 2, 3, or 4 years old), household density (number of people per room in the household, a categorized and continuous variable), number of siblings (none, 1, or more), monthly family income dichotomized into minimum salaries (< 2 or ≥ 2 minimum salaries), the parents’ age (≤ 30 or > 30 years), and whether the child attends school (yes or no—school frequency). In addition, the *Critério Brasil* (“Brazilian Criteria”) questionnaire was applied [21]. This is a standardized questionnaire devised by the Brazilian federal government to collect socio-economic information for statistical purposes. It seeks to classify the consumption potential of Brazilian households into levels. It is based on assets ownership and not on family income. For all possessions, there is a score, and each class is defined by the sum of this score (A, B1, B2, C1, C2, D–E).

The children’s parents or caregivers' OHL level was evaluated by recognizing words included in BREALD-30 [22], the Brazilian adaptation of the 30-word version of the Rapid Estimation of Adult Literacy in Dentistry (REALD-30) OHL test. The questionnaire contains 30 words related to oral diseases (etiology, anatomy, prevention, and treatment) that must be read aloud by the research subjects to the interviewers. Every time the participant pronounces the word correctly, 1 point is awarded. Conversely, every time the participant cannot read the word correctly, no points are awarded. The total score thus obtained ranges from 0 to 30. The highest score corresponds to the highest level of OHL [23]. The score of the lowest quintile (< 13) was defined as the cutoff point that indicates a “low” OHL level.

B-ECOHIS, the Brazilian adaptation of the Early Childhood Oral Health Impact Scale (ECOHIS) [24], was used to measure the OHRQOL of the children in the sample. This questionnaire assesses parents’ perception of their children's OHRQOL and contains 13 questions. Nine of these questions correspond to domains included in the section about the impact on the child: symptom—01 question; function—04 questions; psychology—02 questions; self-image and social interaction—02 questions. The last four questions correspond to domains included in the section about the impact on the family: parental distress—02 questions; family function—02 questions. The answers to each B-ECOHIS question were categorized and coded: 0 = never; 1 = almost never; 2 = sometimes; 3 = often; 4 = very often; 5 = unknown. The B-ECOHIS scores, both total and by domains, were calculated from the sum of the answer codes, and the “unknown” answers were excluded from the analysis.

### Clinical examination

The clinical examination was conducted after the data collection and before the child was vaccinated, thus avoiding manipulating the child's oral cavity after receiving a vaccine in oral drops. Dental caries were diagnosed in deciduous **t**eeth that were **d**ecayed, **m**issing due to extraction, or **f**illed (dmft), as defined by the World Health Organization [25]. Clinical assessment was performed using a systematic approach by quadrant. To assess dental caries, each examiner collected data from 36 children, 12 in each of the three age groups in the sample.

### Statistical analysis

**Fig. 1:**
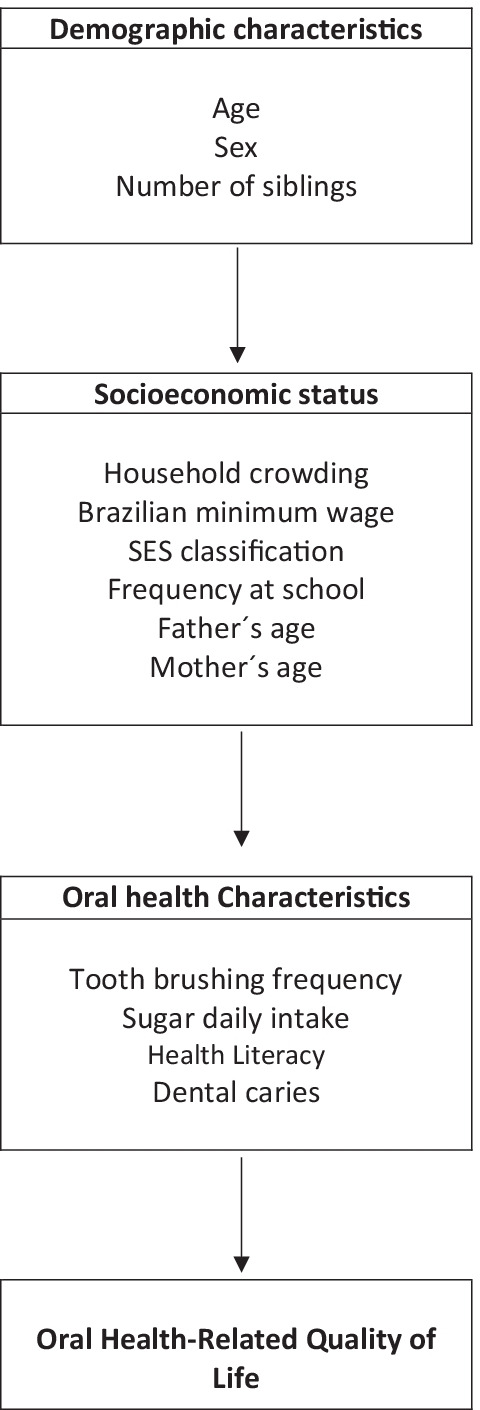
Conceptual hierarchical framework of factors related to oral health-related quality of life

Stata software (StataCorp, College Station, Texas, USA) version 12.0 was used for statistical analysis of the data. Initially, descriptive analyses were performed to obtain all variables' frequency distribution and the prevalence (frequency) and severity (mean and standard deviation) of the domain-specific and total B-ECOHIS score. Unadjusted and adjusted zero-inflated negative binomial (ZINB) regression analysis was used to assess the association between the outcome (OHRQOL) and the prevalence of dental caries, OHL, and other factors. OHRQOL was analyzed as a counting variable (severity) using rate ratios (RR) and as a dichotomous variable (prevalence) using prevalence ratios (PR). The multivariable model followed a conceptual framework (Fig. 1) to adjust prevalence and rate ratios, and their respective 95% Confidence Intervals (95% CI). For the ZINB analyses, the adjusted model was built with covariates chosen by the unadjusted analysis. Covariates of the unadjusted analysis with *p* < 0.20 were considered in the final adjusted model and covariates with *p* < 0.05 were selected to remain in this model [26]. The zero-inflated negative binomial (ZINB) regression was selected due to overdispersion to the left, i.e., the excessive number of zeros in the distribution of the outcome variable.

## Results

As a result of the calibration process for the dental caries examination, a kappa statistic of 0.98 was obtained for intra-examiner agreement and 0.85 for inter-examiner agreement. A total of 630 children and their respective parents or caregivers participated in the study; their average age was 2.98 (0.03, standard deviation) and 332 children (52.7%) were boys. According to the dmft index, 24% of the children had at least one tooth affected by dental caries. According to their parents or caregivers, 80.79% of the preschoolers brushed their teeth two or more times a day, and 71.75% attended school. Regarding mothers’ education, 63% had the equivalent of 10 years of education or more, and 85% of the parents or caregivers showed a score considered ideal for BREALD-30 (above 13 points) (Table 1). All parents interviewed in the study completed the B-ECOHIS and BREALD-30 questionnaires (100% response rate).

**Table 1:** Unadjusted analysis of variables associated with the total score and each domain of B-ECOHIS (prevalence)

Prevalence	n (%)	The child impact section		The family impact section		Score Total B-ECOHIS	
		PR* (95% IC)	*p*	PR* (95% IC)	*p*	PR* (95% IC)	*p*
*Demographic characteristics*							
Age							
2 years	213 (33.81)	Ref					
3 years	213 (33.81)	1.20 (0.82–1.76)	0.33	1.4 (0.78–2.48)	0.25	1.29 (0.91–1.85)	0.15
4 years	204 (32.38)	1.11 (0.75–1.64)	0.61	1.04 (0.56–1.94)	0.89	1.16 (0.81–1.67)	0.43
Sex							
Male	332 (52.70)	Ref					
Female	298 (47.30)	0.86 (0.63–1.18)	0.37	0.78 (0.48–1.26)	0.31	0.89 (0.67–1.19)	0.45
Number of siblings							
None	272 (43.17)	Ref					
1 or more	343 (54.44)	0.78 (0.57–1.07)	0.13	0.79 (0.48–1.28)	0.34	0.77 (0.57–1.04)	0.09
*Socioeconomic characteristics*							
Household crowding							
≤ 1	355 (56.35)	Ref					
> 1	244 (38.73)	0.83 (0.59–1.16)	0.28	1.02 (0.62–1.69)	0.93	0.87 (0.64–1.19)	0.39
Family income							
Up to 2 minimum wage	315 (50.00)	Ref					
≥ 2 minimum wage	296 (46.98)	0.99 (0.72–1.37)	0.98	1.09 (0.68–1.77)	0.70	0.96 (0.71–1.29)	0.79
Mother’s age							
≤ 30 years	312 (49.68)						
> 30 years	314 (50.00)	0.71 (0.52–0.97)	0.03	0.80 (0.49–1.30)	0.38	0.79 (0.59–1.07)	0.13
Mother’s level of education							
≤ 10 years	219 (34.76)	Ref					
> 10 years	401 (63.65)	1.04 (0.75–1.45)	0.81	1.45 (0.84–2.50)	0.17	1.11 (0.81–1.51)	0.51
Father’s age							
≤ 30 years	198 (31.43)	Ref					
> 30 years	382 (60.63)	0.92 (0.66–1.30)	0.66	1.37 (0.78–2.39)	0.26	1.03 (0.75–1.43)	0.82
School frequency**							
Yes	452 (71.75)	Ref					
No	154 (24.44)	0.96 (0.67–1.39)	0.87	0.70 (0.38–1.29)	0.26	0.85 (0.59–1.21)	0.37
*Oral health characteristics*							
Frequency of consumption of sugar							
< 3 vezes times a day	260 (41.27)	Ref					
> 3 times a day	244 (38.73)	1.26 (0.91–1.74)	0.16	1.01 (0.63–1.65)	0.94	1.23 (0.92–1.66)	0.17
Tooth brushing							
≥ 2 a day	509 (80.79)	Ref					
< 2 a day	106 (16.83)	1.20 (0.81–1.78)	0.36	1.50 (0.86–2.64)	0.15	1.19 (0.77–1.62)	0.56
Socioeconomic stratum							
A + B1	200 (31.75)	Ref					
B2 + C1 + C2 + D-E	429 (68.10)	1.39 (0.97–2.00)	0.06	1.04 (0.62–1.74)	0.87	1.18 (0.85–1.63)	0.30
Breald							
Low	90 (14.29)	Ref					
Ideal	538 (85.40)	0.99 (0.64–1.54)	0.98	1.73 (0.74–3.99)	0.2	1.11 (0.72–1.71)	0.62
Untreated caries experience							
Absence	479 (76.04)	Ref					
Presence	151 (23.97)	1.52 (1.09–2.12)	0.01	4.08 (2.49–6.48)	0.00	1.73 (1.28–2.34)	0.00

*PR Prevalence Ratio

**: *Differentiating children that go to school from those that do not

***: Minimum wage: R$ 937,00 (U$ 246.51)

Table 2 shows the prevalence and severity of impact from B-ECOHIS by domains and their respective sections (child and family) and the total score. According to the parents' perception, low prevalence and severity of impact on the children’s quality of life were observed in all domains. The prevalence was 29.2% in the total score (total B-ECOHIS scores > 0), and the domain-specific highest impact was “oral symptoms” (11.4%).

**Table 2:** Prevalence and severity of impact on children's quality of life according to domains and total score

	Prevalence n (%)	Severity Mean (SD)
*The child impact section*		
Child symptom	72 (11.43)	0.13 (0.19)
Child function	71 (11.27)	0.18 (0.03)
Child psychology	70 (11.11)	0.12 (0.02)
Self-image/social interaction	6 (0.95)	0.01 (0.00)
*The family impact section*		
Parent distress	45 (7.14)	0.13 (0.03)
Family function	28 (4.44)	0.04 (0.01)
*Escore Total B-ECOHIS*	184 (29.21)	0.29 (0.02)

In the unadjusted analysis of the negative binomial regression, a negative impact of dental caries on the OHRQOL of children was observed in the prevalence, as well as a positive impact of an older age of the mother in the section “impact on the child” (*p* < 0.05). For severity, there was a significant association between the child having one or more siblings and all sections of B-ECOHIS, as well as dental caries in the section “impact on the child” and the total B-ECOHIS score (*p* < 0.05) (Table 3).

**Table 3:** Unadjusted analysis of variables associated with the total score and each domain of B-ECOHIS (severity)

Severity	The child impact section		The family impact section		Score Total B-ECOHIS	
	RR* (95% IC)	*p*	RR* (95% IC)	*p*	RR* (95% IC)	*p*
*Demographic characteristics*						
Age						
2 years	Ref					
3 years	1.29 (0.79–2.11)	0.30	1.23 (0.73–2.08)	0.42	1.21 (0.80–1.83)	0.36
4 years	0.92 (0.54–1.56)	0.77	1.42 (0.82–2.46)	0.20	1.02 (0.66–1.57)	0.92
Sex						
Male	Ref					
Female	1.38 (0.92–2.06)	0.12	1.35 (0.91–2.01)	0.14	1.21 (0.86–1.68)	0.27
Number of siblings						
None						
1 or more	1.77 (1.19–2.65)	0.00	1.55 (1.03–2.33)	0.03	1.65 (1.19–2.29)	0.00
*Socioeconomic characteristics*						
Household crowding						
≤ 1	Ref					
> 1	1.27 (0.74–1.72)	0.58	0.86 (0.55–1.34)	0.52	0.99 (0.69–1.42)	0.97
Family income						
Up to 2 minimum wage	Ref					
≥ 2 minimum wage	0.77 (0.51–1.14)	0.19	1.12 (0.74–1.68)	0.59	0.97 (0.69–1.36)	0.87
Mother’s age						
≤ 30 years	Ref					
> 30 years	1.33 (0.88–2.00)	0.17	0.95 (0.63–1.43)	0.81	1.12 (0.80–1.56)	0.51
Mother’s level of education						
≤ 10 years						
> 10 years	0.98 (0.64–1.52)	0.95	1.23 (0.74–2.01)	0.42	1.08 (0.75–1.55)	0.66
Father’s age						
≤ 30 years	Ref					
> 30 years	1.08 (0.69–1.68)	0.73	1.22 (0.73–2.04)	0.44	1.16 (0.79–1.68)	0.44
School frequency						
Yes						
No	1.21 (0.76–1.94)	0.41	0.86 (0.49–1.51)	0.61	1.11 (0.74–1.67)	0.58
*Oral health characteristics*						
Frequency of consumption of sugar						
< 3 times a day	Ref					
> 3 times a day	1.04 (0.68–1.60)	0.83	1.32 (0.86–2.04)	0.19	1.13 (0.79–1.59)	0.49
Tooth brushing						
≥ 2 times a day	Ref					
< 2 times a day	0.96 (0.57–1.59)	0.87	1.21 (0.77–1.91)	0.40	1.19 (0.78–1.81)	0.41
Socioeconomic stratum						
A + B1	Ref					
B2 + C1 + C2 + D-E	1.11 (0.68–1.78)	0.67	0.96 (0.63–1.48)	0.87	1.24 (0.85–1.81)	0.26
Breald						
Low	Ref					
Ideal	0.84 (0.47–1.49)	0.56	1.31 (0.59–2.91)	0.50	0.98 (0.60–1.62)	0.96
Untreated caries experience						
Absence	Ref					
Presence	1.63 (1.08–2.47)	0.02	0.99 (0.66–1.50)	0.99	1.66 (1.19–2.29)	0.00

*RR Rate Ratio

In the adjusted model, a higher caries experience was associated with a higher prevalence of impact on quality of life in all sections and in the total B-ECOHIS score (PR = 1.84; 95% CI 1.35–2.50). In addition, there was an association indicating a protective effect of the age of the mother on the section “impact on the child” (PR = 0.72; 95% CI 0.52–0.98), and a similar effect of the variable number of siblings (one sibling or more) on the total B-ECOHIS score (PR = 0.70; 95% CI 0.52–0.95). Regarding the severity of impact, children with one or more siblings had a negative effect on quality of life (RR = 1.28; 95% CI 0.98–1.67). It was also observed that dental caries remained associated with the severity of the impact on the total B-ECOHIS score (PR = 1.42; 95% CI 1.09–1.85) (Table 4). The other factors showed no significant association with the outcome. Thus, no association was observed between OHL and the impact on the children's quality of life.

**Table 4:** Adjusted analysis of variables associated with the total score and each domain of B-ECOHIS

Prevalence	The child impact section		The family impact section		Score Total B-ECOHIS	
	PR* (95% IC)	*p*	PR* (95% IC)	*p*	PR* (95% IC)	*p*
*Number of siblings*						
None	Ref					
1 or more					0.70 (0.52–0.95)	0.02
*Mother’s age*						
≤ 30 years	Ref					
> 30 years	0.72 (0.52–0.98)	0.04				
*Untreated caries experience*						
Absence	Ref					
Presence	1.53 (1.09–2.13)	0.01	4.02 (2.49–6.48)	0.00	1.84 (1.35–2.50)	0.00

Severity	The child impact section		The family impact section		Score Total B-ECOHIS	
	RR** (95% IC)	*p*	RR** (95% IC)	*p*	RR** (95% IC)	*p*
*Number of siblings*						
None	Ref					
1 or more	1.62 (1.05–2.49)	0.02	1.55 (1.03–2.33)	0.03	1.28 (0.98–1.67)	0.06
*Untreated caries experience*						
Absence	Ref					
Presence	1.33 (0.87–2.03)	0.18			1.42 (1.09–1.85)	0.00

*PR Prevalence Ratio

**RR Rate Ratio

## Discussion

The present study found that the OHL of parents and caregivers has no statistically significant association with the OHRQOL of preschool-age children. This finding is the most important result of the study, and it is not due to the reduced statistical power of the sample. Although our results did not confirm the study’s initial hypothesis, this study was the first to assess the association of the OHL of parents and caregivers with the OHRQOL of preschool children in Brazil.

OHL is considered a key element in promoting health and preventing oral diseases, as it enables oral health inequalities in individuals with low OHL to be measured. OHL is more than just a way of measuring the ability of individuals to obtain, process, and understand information about oral health. OHL is a powerful tool for empowering individuals in the social context, as it contributes to facilitating access to health services [27]. Recent studies have sought to assess the relationship between literacy and oral conditions, mainly dental caries [28, 29]). In a systematic review conducted by Firmino et al. [30], a weak association was found between OHL and deciduous-tooth caries in children aged 4 to 6 years. Only three cross-sectional studies [7, 11, 31] were found that assessed the relationship between the parents’ level of literacy and the presence of caries in children. Therefore, scientific evidence is still insufficient. The literature is also inconclusive regarding parental literacy and OHRQOL. The review study by Firmino et al. did not find a statistically significant association between studies that investigated parental OHL and OHRQOL.

Regarding the quality of life, there was a negative impact of dental caries on the children's OHRQOL, which corroborates previous studies [32–34].

Children with one or more siblings had a lower prevalence of impact on OHRQOL In the adjusted model. However, having one or more siblings was also associated with the severity of the negative impact on OHRQOL, which seems to be conflicting with the previous finding. We hypothesize that the presumable protective effect of siblings to the prevalence of impacts suggests that parents benefit from the experience of having taken care of their children's oral health. On the other hand, for those children who have a negative OHRQOL impact, the presence of siblings can favor the severity of the impact, in view of possible difficulties faced by their parents in taking care of more than one child; i.e., less time available to be dedicated to each child. This hypothesis could explain the conflicting results obtained, with the variable having one or more siblings acting as a protective factor for the prevalence of impact and simultaneously as an indicator of risk regarding the severity of the impact on OHRQOL.

The present study also found a positive association between the mother's age and the children's OHRQOL. Mothers above 30 years of age appraised as better their children’s oral health-related quality of life. The same result was observed in the literature [35], reinforcing the present findings. A plausible explanation for the mother's age influencing the B-ECOHIS scores would be the insecurity of younger mothers toward their children, negatively interfering in the section “impact on the child.”

An association between family income and the impact on the children's HRQOL was not observed in the present study. Contrariwise, a previous study reported that children from families with higher incomes and parents with a high level of education had better OHRQOL [36]. Our result of the absent association between socioeconomic status and the outcome variable can have been influenced by the relatively high human development index (HDI) of the municipality of Diadema in the Brazilian context. The city ranked 0.757 for the human development index in 2010 [37], which is a high value for the Brazilian context. Also, the absent association between income and the outcome may reflect factors such as the family’s social support network and the personal perception of the oral health condition, factors that are independent of socioeconomic status [38].

A study carried out with data from the Brazilian Family Budget Survey indicated that private dental expenditures are concentrated in families with higher education and income from states with greater economic development [39]. This scenario would contribute to an increase in inequalities in access and use of health services. However, a higher qualification of the public service could attenuate this effect, contributing to explaining the lack of association between OHRQOL and household income in the present study.

Some study limitations should be considered, such as the cross-sectional design, which prevents causal inferences. The purpose of this study was to assess whether there was an association between the OHL of parents and caregivers and the OHRQOL of children. The fact that the BREALD-30 questionnaire only allows the assessment of word recognition and reading ability, which do not necessarily imply knowledge of the meaning of those words, can also be considered a limitation of the present study. In other words, BREALD-30 may overestimate the OHL of parents and caregivers. Thus, future studies with different designs are suggested for a better understanding of the possible contribution of the OHL of parents and caregivers to children's OHRQOL.

## Conclusions

The level of oral health literacy of parents and caregivers was not associated with the oral health-related quality of life of preschool children, while dental caries and a higher number of siblings had a negative impact. A higher education level of the mother had a positive impact on OHRQOL.

## Data Availability

The datasets during and/or analysed during the current study available from the corresponding author on reasonable request.

## Abbreviations

OHRQOL: Oral health-related quality of life
OHL: Oral health literacy
ECOHIS: Early Childhood Oral Health Impact Scale
ZINB: Zero-inflated negative binomial regression
PR: Prevalence Ratios
RR: Rate Ratios
REALD-30: Rapid Estimation of Adult Literacy in Dentistry
HDI: Human development index
